# Molecular Mechanisms of Glutamate Toxicity in Parkinson’s Disease

**DOI:** 10.3389/fnins.2020.585584

**Published:** 2020-11-26

**Authors:** Ji Wang, Fushun Wang, Dongmei Mai, Shaogang Qu

**Affiliations:** ^1^Department of Neurology, Nanfang Hospital, Southern Medical University, Guangzhou, China; ^2^Guangdong-Hong Kong-Macao Greater Bay Area Center for Brain Science and Brain-Inspired Intelligence, Guangzhou, China; ^3^Key Laboratory of Mental Health of the Ministry of Education, Southern Medical University, Guangzhou, China; ^4^Institute of Brain and Psychological Science, Sichuan Normal University, Chengdu, China; ^5^Department of Neurosurgery, Baylor Scott & White Health, Temple, TX, United States

**Keywords:** glutamate, neurotransmitter, excitotoxicity, oxidative glutamate toxicity, immunoexcitotoxicity

## Abstract

Parkinson’s disease (PD) is a common neurodegenerative disease, the pathological features of which include the presence of Lewy bodies and the neurodegeneration of dopaminergic neurons in the substantia nigra pars compacta. However, until recently, research on the pathogenesis and treatment of PD have progressed slowly. Glutamate and dopamine are both important central neurotransmitters in mammals. A lack of enzymatic decomposition of extracellular glutamate results in glutamate accumulating at synapses, which is mainly absorbed by excitatory amino acid transporters (EAATs). Glutamate exerts its physiological effects by binding to and activating ligand-gated ion channels [ionotropic glutamate receptors (iGluRs)] and a class of G-protein-coupled receptors [metabotropic glutamate receptors (mGluRs)]. Timely clearance of glutamate from the synaptic cleft is necessary because high levels of extracellular glutamate overactivate glutamate receptors, resulting in excitotoxic effects in the central nervous system. Additionally, increased concentrations of extracellular glutamate inhibit cystine uptake, leading to glutathione depletion and oxidative glutamate toxicity. Studies have shown that oxidative glutamate toxicity in neurons lacking functional N-methyl-D-aspartate (NMDA) receptors may represent a component of the cellular death pathway induced by excitotoxicity. The association between inflammation and excitotoxicity (i.e., immunoexcitotoxicity) has received increased attention in recent years. Glial activation induces neuroinflammation and can stimulate excessive release of glutamate, which can induce excitotoxicity and, additionally, further exacerbate neuroinflammation. Glutamate, as an important central neurotransmitter, is closely related to the occurrence and development of PD. In this review, we discuss recent progress on elucidating glutamate as a relevant neurotransmitter in PD. Additionally, we summarize the relationship and commonality among glutamate excitotoxicity, oxidative toxicity, and immunoexcitotoxicity in order to posit a holistic view and molecular mechanism of glutamate toxicity in PD.

## Introduction

Glutamate is the most abundant excitatory neurotransmitter in the mammalian brain. It is widely distributed and involved in a variety of functions and metabolic processes in the central nervous system (CNS) (Murrough et al., 2017; Madji Hounoum et al., 2018; Wang et al., 2018). In the CNS, glutamate is present in more than one type of cell and distributed in many subcellular compartments, including the cytoplasm and mitochondria (Nedergaard et al., 2002). In mammals, there is 5–10 mmol glutamate per kilogram of CNS tissue (Butcher et al., 2010), which is higher than levels of virtually all other neurotransmitters. These levels are a thousand times greater than many other important neurotransmitters, such as dopamine, serotonin, and norepinephrine (Sheldon and Robinson, 2007).

PD is a chronic neurodegenerative disease that affects 2–3% of the population over age 65 (Poewe et al., 2017). The main pathological features of PD are the appearance of Lewy bodies and the death of neurons in the basal ganglia [about 70% of dopaminergic neurons in the substantia nigra pars compacta (SNpc)]. This loss of neurons is accompanied by the death of astrocytes, and a significant increase and activation of microglia in the SNpc. PD is characterized by movement symptoms, such as tremors, bradykinesia, rigidity, and postural instability (Jankovic, 2008). Although many factors have been linked to an increased risk of PD—environmental and genetic factors, aging, and exposure to certain pesticides and fungicides—the exact cause of death of dopaminergic neurons remains unknown.

In 1994, Bergman et al. proposed that excessive activation of glutamate receptors in nigrostriatal neurons might lead to cellular death via glutamate excitotoxicity (Bergman et al., 1994; Hassani et al., 1996; Benazzouz et al., 2002; Hutchison et al., 2010). Previous studies have shown that glutamate excitotoxicity may induce the degeneration of dopaminergic neurons and concomitant motor dysfunction in PD (Sonsalla et al., 1998; Meredith et al., 2009). Under pathological conditions, when excessive glutamate is released from the presynaptic membrane or the reuptake function of glutamate is impaired, the extracellular glutamate concentration increases. Activated microglia and reactive astrocytes release large amounts of glutamate (Takeuchi et al., 2006; Wetherington et al., 2008). Too much glutamate induces excessive stimulation of glutamate receptors and increases the concentration of Na^+^ and Ca^2+^ in the cell, which may directly cause neuronal damage and cell death. In this way, increased extracellular glutamate concentration is the basis for the effects of glutamate excitotoxicity.

**Graphical Abstract:**
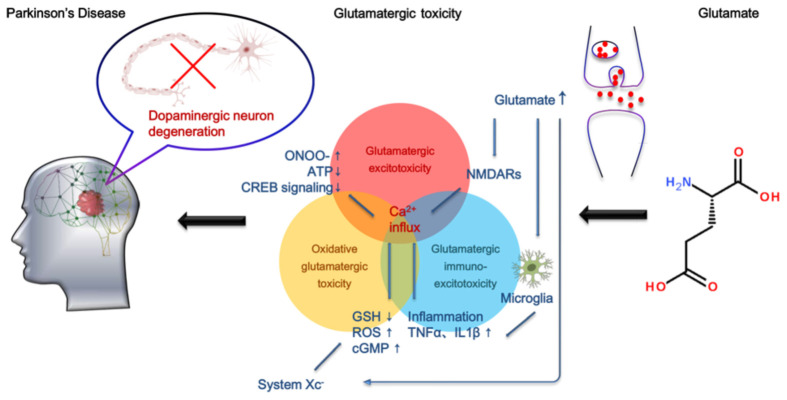
Molecular mechanisms of glutamate toxicity in PD.

## Glutamate

Glucose is a key chemical molecule involved in cell metabolism in the brain; it cannot penetrate the blood–brain barrier (Sibson et al., 1998; Danbolt, 2001), so local synthesis is essential. In the brain, glutamate can be directly synthesized *de novo* by astrocytes or indirectly produced from glucose molecules through the actions of pyruvate dehydrogenase and astrocyte-specific enzyme pyruvate carboxylase (Krzyżanowska Pomierny et al., 2014; Schousboe et al., 2014). Once inside the cell, glucose breaks down through intracellular glucose enzymes to yield acetic acid. Acetic acid enters the tricarboyxlic acid cycle, and then the transfer of ammonia yields alpha-ketoglutarate, which receives an ammonia donor from a chain of amino acids—such as leucine, isoleucine, and valine—and a variety of amino donors, such as aspartic acid, gamma-aminobutyric acid (GABA), and alanine (Luc and Magistretti, 2004). It should be noted that, in addition to acting as a neurotransmitter, glutamic acid also acts as a metabolic precursor of GABA and various amino acid derivatives (Shen et al., 1999). Glutamate taken up by perisynaptic astrocytes is then converted to glutamine via glutamine synthetase (Danbolt, 2001). Astrocytes and neurons contain glutamine transporters, which—under appropriate electrophysiological conditions—result in a net exchange of glutamine between astrocytes and neurons. In neurons, the mitochondrial phosphate-specific enzyme, glutaminase, converts inactive glutamine to glutamate and repackages it into synaptic vesicles. The circulation of glutamate/glutamine in astrocytes and neurons is called the glutamate–glutamine cycle (Niciu et al., 2012). In summary, neuronal glutamate is produced/obtained in two ways: (1) from glucose and amino acid derivatives through energy metabolism; and (2) recovery of glutamate from glutamine via glutamate reuptake (Erecinska and Silver, 1990).

In axons, glutamate is stored in synaptic vesicles; upon sufficient depolarization, glutamate is released via synaptic calcium influx from voltage-gated calcium channels. Glutamate release leads to a significant rise in synaptic glutamate concentrations (∼1,000-fold). Release of presynaptic glutamate and activation of postsynaptic glutamate receptors on the postsynaptic neuron stimulate influx of Na^+^ and Ca^2+^ ions into the postsynaptic neuron, which contributes to depolarization and action potentials (Danbolt, 2001). Astrocytes expressing high levels of excitatory amino acid transporters (EAATs) sequester excess glutamate at synapses (Chaudhry et al., 1995). Glutamate is quickly cleared from the synaptic cleft via glutamate transporters to prevent excessive glutamate-receptor stimulation.

## Glutamate Transporters and Receptors

### Excitatory Amino Acid Transporters

Increased levels of extracellular glutamate, particularly asynchronistic glutamate levels, cause cellular damage when excitatory neurotransmission is sufficiently aberrant (Pitt et al., 2000; Ouardouz et al., 2009). However, the uptake of glutamate at synapses, due to a lack of extracellular enzymes, is achieved primarily through EAATs located in the plasma membranes of neurons and glia (Zhang et al., 2016). Five different EAATs have been identified, and the amino acid sequence homology of these transporters is approximately 50–60% (Sheldon and Robinson, 2007). Based on the crystalline structure of bacterial glutamate transporters (Dinesh et al., 2004), EAATs have eight cross-membrane structures, with a carboxylic acid end and an amino end. It most likely exists as a tripolymer (Yernool et al., 2003; Koch and Peter, 2005). The specific EAAT glutamate aspartate transporter (GLAST) (Storck et al., 1992) is also known as excitatory amino acid transporter 1 (EAAT1) (Arriza et al., 1994), and the EAAT glutamate transporter 1 (GLT-1) (Pines et al., 1992) is also known as excitatory amino acid transporter 2 (EAAT2) (Arriza et al., 1994). GLT-1 and GLAST are mainly found in astrocytes (Rothstein et al., 1994; Lehre et al., 1995). These transporters are also found in many other cells, such as oligodendrocytes and macrophages (Domercq et al., 2010; Gras et al., 2010). Each EAAT has a specific expression pattern, and some of the functional characteristics of EAATs can be attributed to their different spatiotemporal localizations (Furuta et al., 1997b). GLT-1 is not only expressed in astrocytes; its splice variant GLT-1a is also expressed in the axons of hippocampal neurons, which may contribute significantly to glutamate uptake into the axonal terminal (Schmitt et al., 1996; Chen et al., 2004; Berger et al., 2005). Two members of the EAAT family, EAAC1 (also referred to as EAAT3) and EAAT4, are neuronal transporters (Rothstein et al., 1994; Furuta et al., 1997a); in many neurons, however, they appear to be located in postsynaptic elements, indicating that they do not participate in the delivery cycle as monoamine transporters do (Blakely and Bauman, 2000; Torres and Caron, 2003). EAAC1 has been shown to be expressed in hippocampal and cortical pyramidal neurons, as well as in GABAergic neurons (Rothstein et al., 1994), oligodendrocytes (Conti et al., 1998), and other glutamate neurons. The expression of EAAT4 is thought to be limited to cerebellar GABAergic Purkinje cells, but it has also been observed in astrocytes (Wen-Hui et al., 2003). The expression of EAAT5 is confined to retinal rod photoreceptors and bipolar cells (Arriza et al., 1997). About 90% of glutamate transport is mediated by EAAT2. These transporters cooperate with each glutamate (or asparaginase) molecule in the co-transport of two to three Na^+^ ions and a proton and cooperate with the reverse transport of one K^+^ ion (Zerangue and Kavanaugh, 1996). Our previous work has shown that EAATs contribute to the removal of synaptic glutamate, and the protein structures and functions of these transporters are closely related (Shaogang and Kanner, 2008; Crisman et al., 2009; Teichman et al., 2009; Zhang and Qu, 2012; Yunlong et al., 2014; Rong et al., 2015). These findings have helped elucidate the physiological functions of EAATs and their contributions to regulating extracellular glutamate concentrations. However, the role of EAATs in the pathogenesis of various neurodegenerative diseases remains unclear.

### Ionotropic Glutamate Receptors

Glutamate is released in the synaptic cleft and binds to glutamate receptors, which can lead to action potentials. Ionotropic glutamate receptors (iGluRs) are ligand-gated ion channels that are activated by glutamate neurotransmitters, which mediate excitatory synaptic transmission in the CNS; they are the key to synaptic plasticity and play an important role in learning and memory.

According to the selective nomenclature of agonists, the three types of ionotropic receptors are identified as the following: α-amino-3-hydroxy-5-methyl-4-isoxazolepropionic acid receptors (AMPARs), N-methyl-D-aspartic acid receptors (NMDARs), and kainate receptors (KARs). NMDARs consist of a mandatory subunit GluN1 in a combination with GluN2A–2D subunits (Paoletti and Neyton, 2007). AMPARs consist of a combination of GluR1–4 subunits, and KARs are made up of a combination of GluR5–7, KA1, and KA2 subunits (Rosenmund et al., 1998). AMPARs and KARs mainly mediate influx of Na^+^, while NMDARs are doubly gated channels and have high calcium conductivity. Compared with that of other iGluRs, the activity of NMDARs is inhibited via a so-called Mg^2+^ block, which is removed via membrane depolarization (Vargas-Caballero and Robinson, 2004).

NMDARs have the highest affinity for glutamate and are substantially regulated in the mammalian CNS. Activation of synaptic NMDARs can induce neuronal survival through Ca^2+^-mediated signal transduction pathways, whereas overactivation of extrasynaptic pathways can induce neuronal death (Stefanie et al., 2010). Synaptic and extrasynaptic NMDAR activation exert opposing neural protective and neurotoxic effects, respectively, and these processes involve a variety of corresponding neuroprotective and apoptotic pathways (Chai et al., 2001).

### Metabotropic Glutamate Receptors

Glutamate can also activate a type of G-protein-coupled receptors known as metabotropic glutamate receptors (mGluRs). The mGluR family consists of eight subtypes (mGluR1 to mGluR8) that are divided into three groups according to their amino-acid sequences, G-protein coupling, and pharmacological profiles. Together, they participate in the formation of extracellular ligand-binding domains and are responsible for the activation of G-proteins in the hepatic helical transmembrane domain (Rondard and Pin, 2015). Subtypes of Group I include mGluR1 and mGluR5 receptors conjugated with Gq/G11 (Abdulghani et al., 1996; Nicoletti et al., 2011). Group-I mGluRs activate phospholipase C (PLC) to produce inositol 1,4,5 triphosphate (IP3) and diacylglycerol, each of which has multiple second-messenger roles (Nakanishi, 1994; Joly et al., 1995; Pin and Duvoisin, 1995). Group-I mGluRs are mainly located in the post-synaptic density near ionotropic receptors, the function of which is to regulate the excitability of neurons (Shigemoto et al., 1993, 1997). Subtypes of group II (mGluR2, mGluR3) and group III (mGluR4, mGluR6, mGluR7, and mGluR8) are coupled with Gi/Go to activate MAP kinase and PI-3-kinase pathways and negatively regulate adenylate cyclase (Iacovelli et al., 2010; Niswender and Conn, 2010; Nicoletti et al., 2011). These subtypes are located presynaptically and act as self-receptors to inhibit the release of glutamate and/or GABA (Schoepp, 2001). mGluRs have seven transmembrane domains (Platt, 2007), as do other metabotropic receptors, but they are not ion channels. Nevertheless, they activate biochemical cascades that lead to modifications of other proteins. This may lead to changes in synaptic excitability, such as presynaptic inhibition of neurotransmission (Sladeczek et al., 1993) or induction of postsynaptic responses (Chu and Hablitz, 2000; Endoh, 2004; Bonsi et al., 2005; Platt, 2007).

## Glutamate Toxicity

### Glutamate Excitotoxicity

**FIGURE 1:**
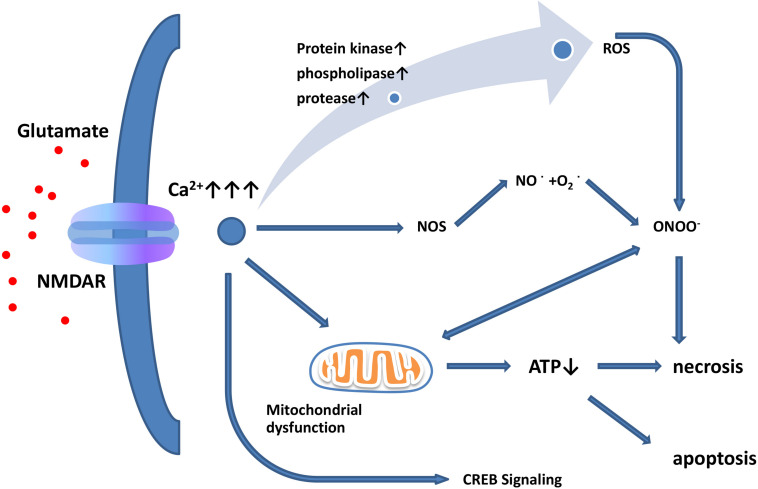
Glutamate excitotoxicity leads to neuronal necrosis and apoptosis. Increased extracellular glutamate levels lead to overactivation of NMDARs and induce Ca^2+^ influx. Ca^2+^ influx increases nitric oxide synthase (NOS) activity. Through this enzyme, NO can react with superoxide radicals to generate ONOO–, thus causing serious oxidative damage to cellular contents. In addition, mitochondria are damaged by oxidation, leading to ATP depletion and cellular death. Excessive activation of extrasynaptic NMDARs leads to reduced CREB signaling, resulting in mitochondrial membrane-potential loss and cell death, while synaptic NMDARs affect the CREB pathway but do not induce apoptosis.

The term “excitatory toxicity” was first proposed in 1986 to describe the ability of excessive extracellular glutamate to kill neurons by activating NMDARs (Choi, 1988; Meldrum and Garthwaite, 1990). Although the mechanisms of excitotoxicity have long been studied, there is still a lack of understanding of the intracellular mechanisms of excitotoxicity leading to neuronal death. When glutamate is released from presynaptic terminals or when glutamate reuptake is impaired, extracellular levels of glutamate increase (Lin et al., 2012). Elevated levels of extracellular glutamate lead to the overactivation of Ca^2+^-permeable NMDARs, subsequent Ca^2+^ overload, and excitotoxicity (Figure 1; Choi, 1988; Bano et al., 2005). In contrast, iGluRs do not have significant permeability to Ca^2+^ (Arundine and Tymianski, 2003; Pinheiro and Mulle, 2006). Under pathological conditions, activated microglia and reactive astrocytes release large amounts of glutamate (Hideyuki et al., 2006; Wetherington et al., 2008). Aberrant high levels of intracellular Ca^2+^ activate catalytic enzymes, produce toxic radicals, and impair production of cellular energy, which ultimately induces cell death (acute necrosis and/or delayed apoptosis; Figure 1; Dutta and Trapp, 2011). Previous studies have shown that this process may activate a variety of enzymes, including kinases, phospholipases, nitric-oxide synthases, and proteases. However, the role of proteases in excitotoxicity remains controversial. The use of calpain inhibitors in hippocampal ischemic neurons was found to reduce the severity of injury (Manev et al., 1990, 2010), but the use of calpain inhibitors did not yield any toxic or protective effect (Manev et al., 2010). In addition, Ca^2+^ influx is not the only factor of excitation-induced cell death. Studies have shown that glutamate exposure or hypoxia/ischemia can trigger the activation of extrasynaptic NMDARs, activate the shutdown of cAMP response element-binding protein (CREB), and lead to mitochondrial membrane-potential loss and cell death (Figure 1). However, the activation of synaptic NMDARs activates only the CREB pathway and does not activate apoptosis (Hardingham and Bading, 2010).

Glutamate-mediated excitotoxicity is involved in many types of neurodegenerative diseases. Currently, there is no safe and effective drug to prevent excitotoxicity. Since overactivation of NMDARs is considered to be the main factor causing glutamate excitotoxicity (Choi, 1987), blocking these receptors is a potential strategy to prevent excitotoxicity. However, most NMDAR antagonists are competitive antagonists that can affect the physiological function of the brain and have negative side effects (Lipton, 2006). Another potential way to prevent excitotoxicity is by enhancing glutamate reuptake. The glial glutamate transporters, EAAT1 and EAAT2, are mainly present in the peripharyngeal processes of astrocytes closely related to excitatory synaptic contact and are responsible for maintaining low levels of extracellular glutamate. EAAT2-knockout mice exhibit fatal spontaneous epilepsy and increased sensitivity to acute cortical injury (Tanaka et al., 1997). In cases of chronic neurodegenerative diseases, such as like PD and Alzheimer’s disease (AD), aberrant EAAT2 function may contribute to excitotoxicity. In other neurodegenerative diseases—such as epilepsy, stroke, and brain trauma—a rapid rise in the levels of extracellular glutamic acid can cause severe neuronal damage. Increasing EAAT2 expression immediately reduces the levels of extracellular glutamate to prevent neuronal damage. This suggests that the up-regulation of EAAT2 may be a potential means of preventing neuroexcitatory toxicity. The expression of EAAT2 in PD animal models has been studied, and the down-regulation of EAAT2 has been found in both 6-hydroxydopamine and acute 1-methyl-4-phenyl-1,2,3, 6-tetrahydropyridine models in the mouse striatum (Holmer et al., 2005; Chung et al., 2010). One study reported that thalidomide can heal the functional damage of nigrostriatal cell substratum by inhibiting excitotoxicity in MPTP mouse models (Palencia et al., 2015). Another study showed that NMDAR antagonists have anti-exercise and anti-dyskinesia effects in Parkinson’s disease (PD) rat models, and they have therapeutic potential in the treatment of PD (Löschmann et al., 2004).

In the pathogenesis of PD, regulation of the glutamate transporter gene is considered to be affected by epigenetic modifications. Epigenetics is currently defined as the study of stable and heritable patterns of gene expression that do not require any changes to the original DNA sequence (Egger et al., 2004; Bird, 2007). Epigenetic modifications mainly include changes in histone modification, DNA methylation, and non-coding RNA. Studies have reported that valproic acid (VPA), which is a histone deacetylase (HDAC) inhibitor can increase the levels of GLAST, GLT-1, and EAAC1 mRNA/protein in astrocytes and oligodendrocytes, thereby combating neurotoxicity (Hassel et al., 2001; Bianchi et al., 2012; Johnson et al., 2018). VPA has different effects on the expression of GLT-1 in different regions of the brain. VPA can increase the expression of GLT-1 in the cortex and hippocampus but decrease expression in the cerebellum (Perisic et al., 2012). This effect may be affected by promoter methylation. Research has indicated that the GLT-1 promoter has multiple methylated CpG islands (Zschocke et al., 2007; Yang et al., 2010), and transcription activation requires demethylation of the GLT-1 promoter. By co-culturing neurons and astrocytes, the methylation of the GLT-1 promoter is reduced (Yang et al., 2010). In the forebrain region, the promoter is hypomethylated and up-regulates the expression of GLT-1, while in the brainstem/cerebellum, the promoter is hypermethylated, while the expression of GLT-1 is not affected (Zschocke et al., 2007). Therefore, GLT-1 expression may be jointly regulated by acetylation and methylation. It has been reported that the increase of miR-543-3p and miR-342-3p in the PD model is associated with decreased expression and function of GLT-1 and increased accumulation of extracellular glutamate and that inhibition of either microRNAs can reverse the effect on the expression and function of GLT-1 and reduce dyskinesia (Wu et al., 2019; Wu et al., 2019).

### Oxidative Glutamate Toxicity

In addition to the receptor-mediated excitotoxicity, an increase in extracellular glutamate can also lead to oxidative stress in the form of the non-receptor-mediated oxidative glutamate toxicity (Tan et al., 2001); this is also the pathway that is induced by glutamate interacting with the Xc^–^ system to produce toxicity. The Xc^–^ system consists of heavy-chain 4F2hc (or CD98/SLC3A2) and light-chain xCT (or SLC7A11), which are connected by disulfide bonds. The light chain of the xCT protein and histone acetyltransferases (HATs) are distinctly homogeneous, and the HATs are a family of amino acid transporters that are linked by the light chain and the heavy chain through disulfide bonds (François et al., 2004). xCT and 4F2hc are both highly expressed in the brain (Kanai et al., 1998). However, it has been shown that the substitution of rBAT for 4F2hc heavy chain does not affect the function of the transporter (Wang et al., 2003; Fernández et al., 2006). The Xc^–^ system is a sodium- and chloride-dependent amino acid antiporter. It transports non-essential sulfur-containing amino acid cysteine anionic compounds into cells and glutamate in a ratio of 1:1 in the opposite direction (Sato et al., 1999; Makowske and Christensen, 2012). Cystine-mediated uptake is critical to cellular antioxidant stress, and xCT plays a major role in maintaining low concentrations of extracellular glutamate and cystine-mediated uptake.

**FIGURE 2:**
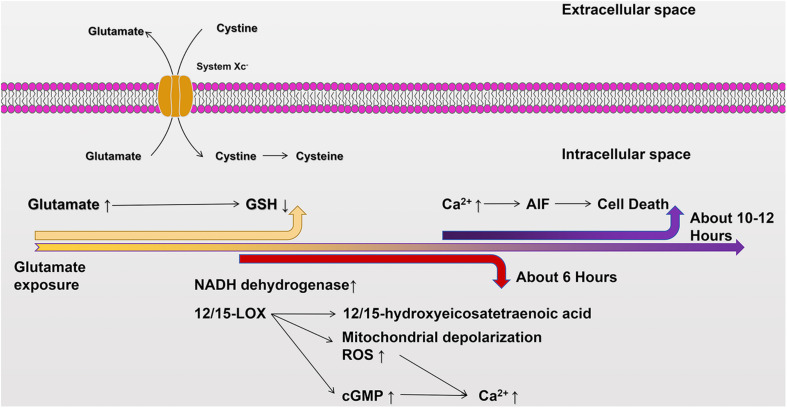
The cellular death pathway of oxidative glutamate toxicity. Excessive accumulation of glutamate in the extracellular environment inhibits cystine uptake through System Xc^–^, resulting in GSH deficiency in cells. Mitochondrial complex I increases about 6 h after glutamate exposure. At the same time, 12/15-LOX is activated and 12/15-hydroxyhexotetraenoic acid is produced, and 12/15-LOX directly damages mitochondria and increases production of ROS. Additionally, soluble functional acid cyclase is activated, and intracellular GMP content is increased. Subsequently, cGMP opens calcium channels and enables calcium influx. After glutamate exposure for about 10–12 h, ROS and intracellular calcium levels reach their peaks, and AIF is transferred from mitochondria to the nucleus, inducing nuclear coagulation and cellular death.

The cell death pathway, which works via oxidative glutamate toxicity, is as follows. The first step in oxidative glutamate toxicity is the inhibition of cystine uptake through the Xc^–^ system leading to GSH deficiency in cells. Although it has been found that some cells can synthesize cystathionine via cystathionine beta-lyase, neurons do not normally express this enzyme and, thus, rely primarily on extracellular cystine. When cystine absorption is blocked, intracellular GSH consumption is much faster than protein synthesis (Tan et al., 1998). In addition, the inhibition of the Xc^–^ system, the subsequent accumulation of ROS, and the decrease of GSH levels create a void in the antioxidant defenses of the cell, which may ultimately lead to ferroptosis (Dixon et al., 2012; Xie et al., 2016). The second step in oxidative glutamate toxicity occurs when the concentration of GSH falls below 20% (about 6 h after glutamate exposure). The continuous generation of free radicals via mitochondrial complex I activity increases exponentially. At this time, the activation of the lipid-oxidizing enzyme, 12/15-lipoxygenase (12/15-LOX), produces 12/15-hydroxyeicosatetraenoic acid (Li et al., 1997a). Third, 12/15-lox can directly damage mitochondria, leading to mitochondrial depolarization and increased the production of reactive oxygen species (ROS) (Stefanie et al., 2010). The eicosanoids produced by 12-LOX are the activators of soluble functional acid cyclase, which can increase intracellular guanosine monophosphate (GMP) (Li et al., 1997b). Elevated cyclic GMP (cGMP) eventually opens calcium channels, leading to harmful influx of calcium (Li et al., 1997b). Finally, oxidative glutamate toxicity induces oxidation of glutamate for approximately 10–12 h. At this time, both ROS and intracellular calcium levels reach maximal values, apoptotic inducing factor (AIF) translocates from mitochondria to nuclei, and intracellular induction of nuclear condensation and caspase-independent cell death occur within a few minutes (Landshamer et al., 2008). This important process is depicted in Figure 2.

### Glutamate Excitotoxicity Cascade in PD

The excitotoxic cascade is triggered by excess extracellular glutamate, which may eventually lead to cell injury and death (Siesjö et al., 1989). Via excessive activation of NMDARs, increased Ca^2+^ influx further exacerbates ROS levels, leads to mitochondrial damage, and increases susceptibility to cellular death. Additionally, excessive activation of AMPARs and KARs induces Na^+^ overload, resulting in high intracellular permeability and acute cellular swelling. The increase of free radicals is particularly important for the pathogenesis of PD, because dopaminergic neurons in the SNpc are particularly susceptible to oxidative stress (Julie and Patrik, 2002; Yuan et al., 2008; Mosharov et al., 2009; Benjamin et al., 2010).

One of the findings of oxidative stress in the SNpc of PD patients is that GSH levels are significantly reduced only in the SNpc, which leads to changes in the GSH/GSSG ratio. In addition, the oxidation of lipids, proteins, and DNA, and the increase of total iron content in the SNpc of PD patients support the enhancement of the oxidation stress (Floor and Wetzel, 1998; Dexter et al., 2010). In this case, oxidative stress in the SNpc leads to excessive peroxide formation, and glutathione deficiency leads to a decreased ability of the brain to remove H_2_O_2_, and continuous accumulation of H_2_O_2_ induces cellular death (Wang et al., 2008; Saggu et al., 2010).

David et al. proposed that the excitotoxicity cascade can be divided into three parts. First, cellular death pathways start with the activation of NMDA receptors, after a brief exposure to a low concentration of glutamic acid. The second part involves 20–30% of the cell death pathway and has characteristics of oxidative glutamate toxicity, because it can be inhibited specifically by vitamin E, group I metabotropic receptor agonists, a caspase inhibitor, elevated extracellular cystine, and the removal of extracellular glutamate (Schubert and Piasecki, 2001). In addition, oxidative glutamate toxicity is higher than the glutamate concentration required for NMDA receptor activation (Murphy et al., 1989). This suggests that oxidative glutamate could be a part of an excited viral cascade and may provide a form of cell death that is independent of iGluRs. Therefore, neurons lacking postsynaptic glutamate receptors are also killed in this process, and oxidative glutamate toxicity may cause more damage than excitotoxicity.

### Pathological Features of PD and Glutamate Toxicity

Another major characteristic of PD is the formation of the Lewy bodies. Lewy bodies are formed by the combination of alpha-synuclein with ubiquitin (Simone, 2008), neurofilament protein, and alpha B crystalline. The Lewy body is thought to be an aggregating response in cells (Mikiei et al., 2004), which suggests that their function may be related to protein folding and aggregation defects (Mikiei et al., 2004; Kovacs, 2015) and may have protective effects on nerve endings (Sreeganga et al., 2005). The typical Lewy body is a postsynaptic cytoplasmic inclusion body consisting of a dense nucleus surrounded by a 10 nm wide ring of radiating fibers. As the main structural component of the Lewy body, alpha-synuclein mainly resides in neurons and is primarily expressed in cerebral cortex, hippocampus, substantia nigra, thalamus, and cerebellum, and is also found in glia. Alpha-synuclein is found in the cerebrospinal fluid of PD patients. In general, the secretion of synuclein in degenerating neurons is greatly increased compared with that of healthy neurons, and alpha-synuclein has enhanced immune characteristics when oxidized or nitrated. Previous studies have shown that alpha-synuclein is closely associated with glutamate excitotoxicity. Watson et al. reported that the relative release of glutamate was inhibited by the knockout of alpha-synuclein in a mouse model in SN terminals (Watson et al., 2009). In a transgenic mouse model of mutant alpha-synuclein (A53T), experiments showed that alpha-synuclein accumulation in astrocytes affected glutamate transport, triggering a rise of extracellular glutamatergic concentrations and excitotoxicity, which further exacerbated damage to dopaminergic neurons (Gu et al., 2010). These findings highlight that alpha-synuclein increases the release of glutamate. Furthermore, some studies have shown that the concentration of alpha-synuclein depends on the release of activity-dependent presynaptic glutamate from forebrain terminals (Sarafian et al., 2017). Additionally, overexpression of alpha-synuclein increases the phosphorylation of NMDARs, thereby increasing the expression of NR1 and NR2B subunits and increasing the sensitivity of NMDARs to glutamate excitotoxicity (Yang et al., 2016). In addition, some studies have found that alpha-synuclein can also enhance glutamate excitotoxicity by accelerating AMPAR signaling (Sandra et al., 2011). The above studies indicate that the abnormal aggregation of alpha-synuclein in PD is closely related to the excitatory toxicity of glutamic acid, which may be a potential neuropathological pathway.

### Glutamate Immunoexcitotoxicity in PD

Extensive microglial activation in and around the SNpc was first observed more than 30 years ago in autopsies of patients with PD (McGeer et al., 1988). It has been reported that macrophages may undergo pathological diffusion from blood vessels to the CNS during inflammation and transform into microglia cells, which promote the pathological development of intracranial nervous system diseases (Chen et al., 2016). Microglia mediating chronic inflammation is involved in the pathological processes of a variety of chronic neurodegenerative diseases. In these processes, microglia are activated over a time span of minutes and can last a long time, or even a lifetime (Ramlackhansingh et al., 2011), which then continuously release a series of inflammatory mediators, leading to oxidative stress. Microglia are activated by the increased production of pro-inflammatory and anti-inflammatory cytokines, chemokines, and other inflammatory molecules, and increased glutamate release, all of which remain in microglia (Hoeijmakers et al., 2016). When the destructive elements are fully activated in microglia, they will show strong inflammatory and excitatory toxic reactions, exceeding the response level of physiological activation of microglia (Norden et al., 2015). In this review, the two most studied neuronal excitatory factors, IL-1β and TNF-α, are discussed as examples. Studies have shown that TNF-α—when used alone in organotypic hippocampal–entorhinal cortex brain slices—causes minor neuronal damage; however, when high-frequency glutamate concentrations are increased, neurotoxicity is greatly accelerated (Zou and Crews, 2005). Additionally, for non-Ca^2+^-permeable AMPA receptors, studies have shown that low-dose TNF-α plays a neuroprotective role through tissue necrosis factor receptor-2 (TNFR-2), while high-dose TNF-α increases the synaptic transport of calcium-permeable AMPARs through the TNFR-1 and enhances excitotoxicity in a concentration-dependent manner (Blaylock, 2017). In addition, it has been demonstrated that the interaction between IL-1β receptors and NMDARs can also lead to increased excitotoxicity, and the expression of IL-1β receptors on the postsynaptic membrane is stimulated by NMDAR activation (Gardoni et al., 2011). As mentioned above, inflammation can change the effects of inflammatory factors, such as TNF-α and IL-1β, and can also enhance glutamine enzymes and inhibit glutamine dehydrogenase and glutamine synthase, thus enhancing glutamate excitotoxicity (Brown and Vilalta, 2015). These two enzymes play crucial roles in glutamate metabolism, and both are sensitive to oxidative stress in the body in an inflammatory environment (Levine, 1983; Aguirre et al., 1989). Glutamine synthase catalyzes the conversion of glutamic acid to glutamine to reduce the risk of excitatory intoxication. Glutamate dehydrogenase, which converts glutamate and ketoglutarate in both directions, also protects against elevated glutamate levels. Conversely, the abovementioned cysteine–glutamic acid reverse transporter (system Xc^–^) can exchange extracellular cystine with intracellular glutamic acid, thus providing the necessary raw materials for the synthesis of glutathione by glial cells; additionally, in the pathological state, it has also become a factor for the excitatory toxicity of glutamic acid. Overall, when inflammation is caused by the activation of microglia, there is a tight connection between inflammation and glutamate receptors. Inflammation can trigger the excessive release of glutamate. Additionally, the accumulation of extracellular glutamate can also strengthen inflammation, forming a vicious cycle (Blaylock, 2017).

## Conclusion

Glutamate affects the CNS to a much greater extent than solely as a CNS courier. In PD, dopaminergic information transmitted by dopaminergic neurons decreases, while glutamatergic signaling in the basal ganglia increases and stimulates the release of dopamine through surviving dopaminergic neurons in the SNpc as a compensatory mechanism. However, by increasing glutamate and excessive activation of glutamate receptors, it could be a “critical strike” to dopaminergic neurons in PD patients. Excessive activation of NMDARs induces excessive influx of Ca^2+^ and further exacerbates ROS levels. Due to the high metabolism of the brain and the characteristics of relatively low regeneration, the brain is sensitive to oxidative damage produced by ROS levels and enhanced oxidative states will also lead to excessive oxidation of lipids, proteins, DNA, and increased total iron content in the SNpc. Overactivation of AMPARs and KARs induces Na^+^ overload, which leads to increased cellular permeability and results in cellular swelling and neuronal death. During abnormal rises in extracellular glutamate, the cystine/glutamate antiporter system Xc^–^ has inhibited cystine transport into cells. Additionally, the ability of glutathione to be reduced by synthesis and the ability of the brain to remove peroxides are compromised; hence, peroxide is accumulated in cells and causes damage, which can also lead to cellular death.

Second, in PD, the abnormal aggregation of alpha-synuclein may increase excitotoxicity by affecting the transport efficiency of glutamate transporters and increasing the phosphorylation of NMDARs. In addition, it is noteworthy that PD microglial cells, after being activated for a short time, will release a series of inflammatory mediators to generate oxidative stress. Neurodegeneration associated with this inflammation is an activated cellular mechanism secondary to signal transduction in inflammatory cells, including lipid peroxidation, reactive oxygen, reactive nitrogen, prostaglandins, proteases, and nitric oxide (Wei et al., 2005). Studies have shown that excitotoxicity may play a major role in the death of dopaminergic neurons during this process.

At present, dopamine replacement therapy is still the gold standard for the treatment of PD, although the efficacy of this therapy is not ideal. Research reports have indicated that Nedd4-2 knockdown attenuates astrogliosis and reactive microgliosis in PD model mice. This may be associated with glutamate excitotoxicity (Zhang et al., 2017). In addition, an antagonist of NMDAR attenuated motor symptoms in an MPTP-induced PD animal model (Pajarillo et al., 2019). Hence, further in-depth research into the different mechanisms of glutamate toxicity or the complexity related to other central neurotransmitters may provide more information for understanding the pathogenesis of PD. Therapeutic intervention to reduce glutamate toxicity will be a possible way to treat PD.

## Author Contributions

SQ: conceptualization and methodology. DM: visualization. SQ, JW, and FW: writing. All authors contributed to the article and approved the submitted version.

## Conflict of Interest

The authors declare that the research was conducted in the absence of any commercial or financial relationships that could be construed as a potential conflict of interest.
